# Using the WHO-INTEGRATE framework to develop a COVID-19 guideline for schools, Germany

**DOI:** 10.2471/BLT.24.291550

**Published:** 2024-08-27

**Authors:** Eva A Rehfuess, Lisa Pfadenhauer, Monika Nothacker, Brigitte Strahwald

**Affiliations:** a Faculty of Medicine, Ludwig-Maximilians-Universität München, Elisabeth-Winterhalter-Weg 6, 81377 Munich, Germany.; b Association of the Scientific Medical Societies in Germany, Berlin, Germany.

## Abstract

**Problem:**

At the beginning of the coronavirus disease 2019 (COVID-19) pandemic, reliable, globally applicable recommendations for safe and continuous school operations were lacking.

**Approach:**

In October 2020, the German Association of Scientific Medical Societies’ task force for COVID-19 guidelines and public health researchers at Ludwig-Maximilians-Universität München initiated the rapid development of a living evidence- and consensus-based guideline to reduce severe acute respiratory syndrome coronavirus 2 transmission in schools. To facilitate transparent, structured and comprehensive decision-making with a whole-of-society perspective, they applied the WHO-INTEGRATE evidence-to-decision framework. This framework supported a broad, multisectoral composition of the guideline panel. The panel used newly synthesized evidence on nine school measures. Participating medical societies or the guideline secretariat completed evidence-to-decision tables. They also drafted recommendations for the guideline panel, who discussed and revised them during moderated consensus conferences.

**Local setting:**

In Germany, each state is responsible for organizing schooling. The German Association of Scientific Medical Societies coordinates development of evidence- and consensus-based guidelines.

**Relevant changes:**

The first version of the guideline was published in February 2021, and the guideline dissemination created much media attention. Of the 16 state education ministries, almost all knew about the guideline, nine recognized it as a relevant source of information and five used it to check existing directives.

**Lessons learnt:**

The WHO-INTEGRATE framework facilitated a comprehensive assessment of school measures from the start of guideline development, considering the broad societal impact of the measures. Using the framework in rapid mode was feasible, but it fell short of its potential.

## Résumé

**Problème:**

Au début de la pandémie de maladie à coronavirus 2019 (COVID-19), il n'existait pas de recommandations fiables et applicables à l'échelle mondiale pour assurer le fonctionnement des écoles sans interruption et en toute sécurité.

**Approche:**

En octobre 2020, le groupe de travail de l'Association allemande des sociétés scientifiques et médicales chargé d'émettre des lignes directrices sur la COVID-19, en collaboration avec des chercheurs en santé publique de l'Université Louis-et-Maximilien de Munich, a lancé le développement rapide d'une orientation évolutive fondée sur le consensus et sur des preuves, afin de limiter la transmission du coronavirus 2 du syndrome respiratoire aigu sévère dans les écoles. Pour favoriser un processus décisionnel transparent, structuré et exhaustif adoptant la perspective de la société dans son ensemble, il a appliqué le cadre WHO-INTEGRATE, un outil privilégiant une prise de décision fondée sur des données probantes. Ce cadre a permis de composer un vaste panel multisectoriel qui, à la lumière d'une synthèse récente d'éléments factuels, a examiné neuf mesures scolaires. De leur côté, les sociétés médicales participantes ou le Secrétariat des directives ont complété des tableaux de prises de décision fondées sur des données probantes. Ils ont également formulé des recommandations à l'attention du panel, qui en a discuté et les a révisées lors de conférences de concertation modérées.

**Environnement local:**

En Allemagne, l'éducation relève de la responsabilité des États fédérés. C'est l'Association allemande des sociétés scientifiques et médicales qui coordonne l'élaboration de lignes directrices fondées sur le consensus et sur des preuves.

**Changements significatifs:**

La première version de l'orientation a été publiée en février 2021 et sa diffusion a engendré une forte couverture médiatique. Parmi les 16 Ministères de l'Éducation des États fédérés, presque tous en connaissaient l'existence, neuf d'entre eux la considéraient comme une source d'information pertinente et cinq l'utilisaient pour vérifier les directives existantes.

**Leçons tirées:**

Vu l'impact majeur de ces mesures sur la société, le cadre WHO-INTEGRATE a contribué à une évaluation globale des mesures scolaires dès le début de l'élaboration de l'orientation. Ce cadre a pu être rapidement mis en œuvre, mais n'a pas atteint son plein potentiel.

## Resumen

**Situación:**

Al principio de la pandemia de la enfermedad por coronavirus de 2019 (COVID-19), no existían recomendaciones fiables y aplicables a escala mundial para el funcionamiento seguro y continuo de las escuelas.

**Enfoque:**

En octubre de 2020, el grupo de trabajo encargado de las directrices sobre la COVID-19 de la Asociación alemana de sociedades científicas médicas e investigadores de salud pública de la Ludwig-Maximilians-Universität München iniciaron el desarrollo rápido de una directriz activa basada en la evidencia y el consenso para reducir la transmisión del síndrome respiratorio agudo grave por coronavirus 2 en las escuelas. Para facilitar una toma de decisiones transparente, estructurada y exhaustiva con una perspectiva de toda la sociedad, aplicaron el marco WHO-INTEGRATE, una herramienta para la toma de decisiones basada en evidencias. Este marco propició una composición amplia y multisectorial del panel de directrices. El panel utilizó pruebas recientemente sintetizadas sobre nueve medidas escolares. Las sociedades médicas participantes o la secretaría de la directriz completaron las tablas de evidencia para la toma de decisiones. También redactaron recomendaciones para el panel de directrices, que las debatió y revisó durante conferencias de consenso moderadas.

**Marco regional:**

En Alemania, cada estado es responsable de organizar la escolarización. La Asociación alemana de sociedades científicas médicas coordina la elaboración de directrices basadas en la evidencia y el consenso.

**Cambios importantes:**

La primera versión de la directriz se publicó en febrero de 2021 y su difusión suscitó una gran atención mediática. De los 16 ministerios de educación estatales, casi todos conocían la directriz, nueve la reconocieron como fuente de información pertinente y cinco la utilizaron para verificar las directrices existentes.

**Lecciones aprendidas:**

El marco WHO-INTEGRATE permitió una evaluación exhaustiva de las medidas escolares desde el inicio de la elaboración de las directrices, y tuvo en cuenta el amplio impacto social de las medidas. Utilizar el marco en modo rápido fue factible, pero no alcanzó su potencial.

## ملخص

**المشكلة:**

في بداية جائحة مرض فيروس كورونا 2019 (كوفيد 19)، كانت هناك حاجة لتوصيات موثوقة، وقابلة للتطبيق عالميًا من أجل عمليات مدرسية آمنة ومستمرة.

**الأسلوب:**

في أكتوبر/تشرين أول 2020، بدأ فريق العمل التابع الهيئة الألمانية للجمعيات الطبية العلمية، والمعنية بالمبادئ التوجيهية لكوفيد 19، والباحثين في مجال الصحة العامة في جامعة لودفيج ماكسيميليانز في ميونيخ، في التطوير السريع لمبادئ توجيهية مباشرة تعتمد على الأدلة والإجماع، وذلك بهدف الحد من انتشار فيروس كورونا 2 المسبب لمتلازمة الجهاز التنفسي الحادة الشديدة في المدارس. لتسهيل اتخاذ قرارات تتمتع بالشفافية والتنظيم والشمول، من منظور المجتمع بالكامل، فقد قاموا بتطبيق إطار عمل WHO-INTEGRATE بدءًا من الأدلة وحتى القرار. دعم إطار العمل هذا تكوينًا واسع النطاق ومتعدد القطاعات للجنة المبادئ التوجيهية. استخدمت اللجنة أدلة تم تجميعها حديثًا حول تسعة من الإجراءات المدرسية. أكملت الجمعيات الطبية المشاركة أو أمانة المبادئ التوجيهية جداول من الأدلة وحتى القرار. كما قاموا أيضًا بصياغة التوصيات للجنة المبادئ التوجيهية، والتي قامت بمناقشتها ومراجعتها أثناء مؤتمرات الإجماع المتوسطة.

**المواقع المحلية:**

في ألمانيا، تعد كل ولاية مسؤولة عن تنظيم العمل في المدارس. تقوم الهيئة الألمانية للجمعيات الطبية العلمية بتنسيق تطوير المبادئ التوجيهية القائمة على الأدلة والإجماع.

**التغيّرات ذات الصلة:**

نُشرت النسخة الأولى من المبادئ التوجيهية في فبراير/شباط 2021، وجذب نشر المبادئ التوجيهية اهتمامًا إعلاميًا كبيرًا. من بين وزارات التعليم الحكومية الست عشرة، كانت جميعها تقريبًا على علم بالمبادئ التوجيهية، وأقرت تسع وزارات بأنها مصدر مهم للمعلومات، واستخدمتها خمس وزارات للتحقق من التوجيهات الحالية.

**الدروس المستفادة:**

أدى إطار عمل WHO-INTEGRATE إلى تسهيل إجراء تقييم شامل لإجراءات المدارس منذ بداية تطوير المبادئ التوجيهية، مع مراعاة التأثير المجتمعي الواسع لهذه الإجراءات. كان استخدام إطار العمل في وضع سريع ممكنًا، لكنه عجز عن تحقيق إمكاناته.

## 摘要

**问题:**

在新冠病毒 (COVID-19) 疫情之初，缺乏适用于全球的可确保学校安全和持续运行的可靠建议。

**方法:**

2020 年 10 月，德国科学医学会协会的 COVID-19 指南特别工作组和慕尼黑大学的公共卫生研究人员着手快速制定了一份基于实时证据和共识的指南，以减少严重急性呼吸系统综合症冠状病毒 2 在学校的传播。为了从全社会的角度提升决策的透明化、结构化和全面性，他们实施了 WHO-INTEGRATE 循证决策框架。该框架支持由广泛、多部门组成的指南小组。该小组利用最新的综合证据制定了九项学校措施。参与的医学会或指南小组秘书处编制了循证决策表格。他们还为指南小组起草了建议，该小组在所主持的共识会议上讨论并修改了这些建议。

**当地状况:**

在德国，每个州都负责组织学校教育。德国科学医学会协会负责协调并制定基于证据和共识的指南。

**相关变化:**

第一版指南于 2021 年 2 月发布，该指南的传播引起了媒体的广泛关注。在 16 个州的教育部中，几乎所有人都知道该指南，其中 9 个州认为它是相关信息的一个来源，5 个州用它来检查现有的方针。

**经验教训:**

鉴于这些措施产生的广泛社会影响，WHO-INTEGRATE 框架从指南制定之初就促进了对学校措施的全面评估。快速利用该框架制定指南是可行的，但未能充分发挥其潜力。

## Резюме

**Проблема:**

В начале пандемии коронавирусной инфекции 2019 года (COVID-19) отсутствовали надежные, применимые во всем мире рекомендации по обеспечению безопасной и бесперебойной работы школ.

**Подход:**

В октябре 2020 года целевая группа Немецкой ассоциации научных медицинских обществ по разработке рекомендаций по борьбе с COVID-19 и исследователи в области общественного здравоохранения из Университета Людвига-Максимилиана в Мюнхене приступили к оперативной разработке основанного на фактических данных и консенсусе руководства по снижению передачи коронавируса тяжелого острого респираторного синдрома 2-го типа в школах. Для содействия прозрачному, структурированному и всеобъемлющему принятию решений с учетом интересов всего общества они применили разработанную ВОЗ систему учета фактических данных при принятии решений WHO-INTEGRATE. Эта структура поддерживала широкий, многосекторальный состав группы по разработке руководящих принципов. Группа использовала новые обобщенные данные по девяти школьным показателям. Участвующие медицинские общества или секретариат группы разработки руководства заполнили таблицы фактических данных, необходимые для принятия решения. Они также подготовили проект рекомендаций для группы экспертов, которые обсудили и пересмотрели их в ходе модерируемых конференций по достижению консенсуса.

**Местные условия:**

В Германии каждая земля несет ответственность за организацию школьного образования. Немецкая ассоциация научных медицинских обществ координирует разработку руководящих принципов, основанных на фактических данных и консенсусе.

**Осуществленные перемены:**

Первая версия руководства была опубликована в феврале 2021 года, и распространение руководства привлекло большое внимание средств массовой информации. Из 16 земельных министерств образования почти все были осведомлены об этом руководстве, девять признали его важным источником информации, а пять использовали для проверки существующих директив.

**Выводы:**

Рамочная структура WHO-INTEGRATE способствовала всесторонней оценке школьных мер с самого начала разработки руководящих принципов с учетом их широкого влияния на общество. Использование системы в ускоренном режиме было возможным, но ее потенциал оказался недостаточным.

## Introduction

Public health and social measures played a pivotal role in containing the coronavirus disease 2019 (COVID-19) pandemic. Most governments closed schools during the early months of the pandemic.^1^ However, the evidence base regarding the effectiveness^2^ and unintended consequences of school-based measures^3^ was evolving and uncertain, and globally applicable evidence-based guidelines were not available.

In Germany, all schools fully closed for at least nine weeks in spring 2020 and were partially closed for up to 24 weeks in winter 2020/2021.^4^ When they re-opened, schools implemented various combinations of school-based public health and social measures, including hybrid learning, the use of face masks by students and teachers, and ventilating classrooms, to reduce severe acute respiratory syndrome coronavirus 2 (SARS-CoV-2) transmission. Each of Germany’s 16 states developed and updated its own directives for primary and secondary schools. The acceptability of these measures varied widely among students, teachers and school personnel, parents and the population at large. 

Therefore, to reduce the risk of infection and to enable safe and continuous school operations, reliable, nationally applicable recommendations on school measures were needed.

## Local setting

Governance in Germany comprises the federal government and 16 states, with each state’s education ministry responsible for organizing the schooling. 

The German Association of Scientific Medical Societies coordinates development of evidence- and consensus-based guidelines, produced according to international standards.^5^ While the association’s clinical guidelines are well established, the development of public health guidelines is still at an early stage.

## Approach

In response to the need for reliable recommendations, the rapid development of a living evidence- and consensus-based guideline to prevent and control SARS-CoV-2 transmission in schools started in October 2020.

**Fig. 1:**
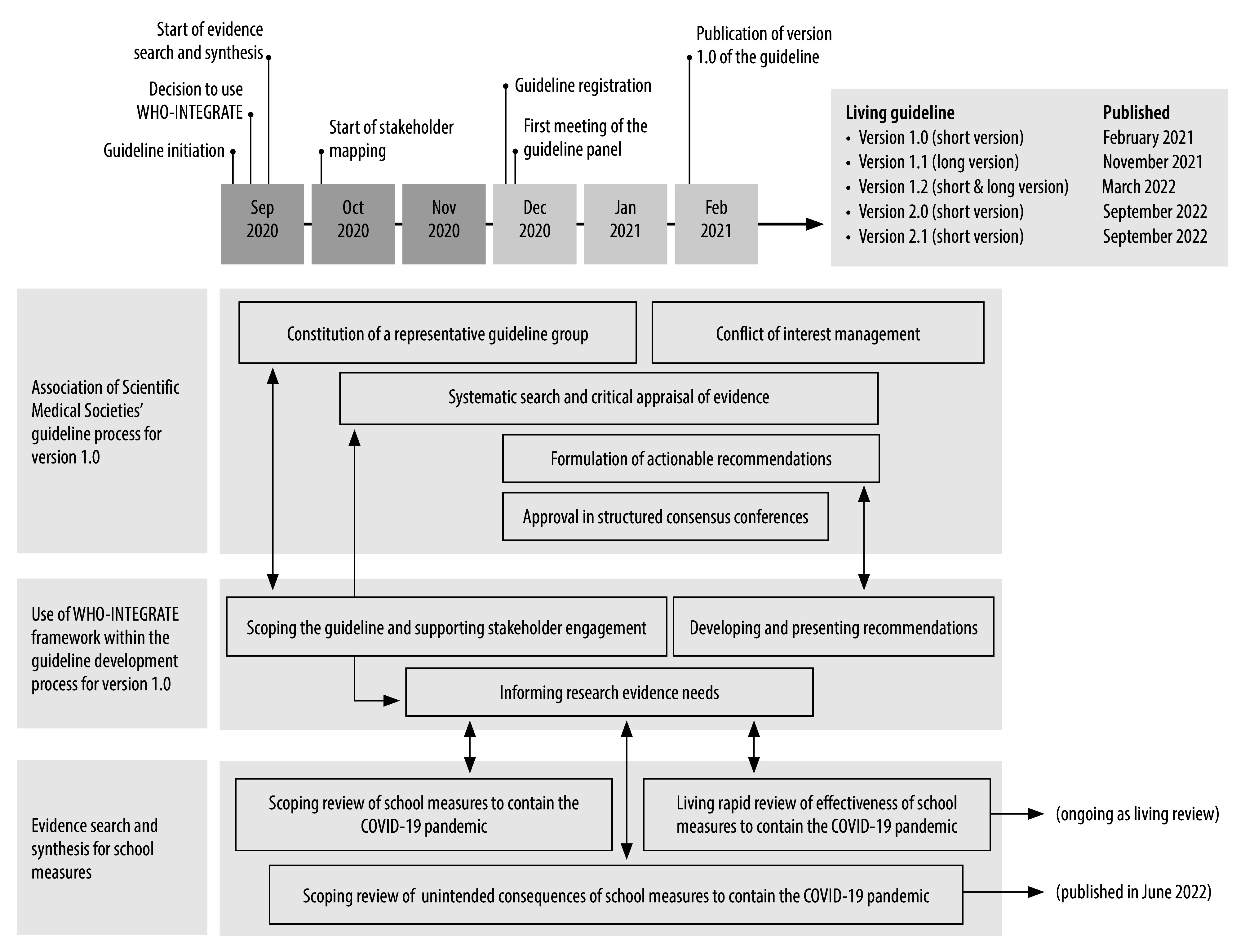
Timeline of the rapid development process of the *S3 guideline – measures for the prevention and control of SARS-CoV-2 transmission in schools – living guideline*, Germany, 2020–2022

COVID-19: coronavirus disease 2019; WHO: World Health Organization

Note: the figure emphasizes the role of the WHO-INTEGRATE framework in the guideline development process.

The Association of Scientific Medical Societies’ task force for COVID-19 guidelines, together with public health researchers at Ludwig-Maximilians-Universität München initiated the development in the context of the COVID-19 evidence ecosystem (CEOsys) project.^6^ The CEOsys project provided the financial resources to conduct multiple Cochrane reviews (Fig. 1).^2^^,^^3^^,^^7^ The Chair of Public Health and Health Services Research at Ludwig-Maximilians-Universität München allocated several full-time staff to host the guideline secretariat and the Association of Scientific Medical Societies, as the coordinating body, offered methodological support. All 32 actively involved organizations listed in Box 1 contributed significant staff time.

Box 1 **Composition of the guideline panel developing the *S3 guideline – measures for the prevention and control of SARS-CoV-2 transmission in schools – living guideline*, Germany, 2020–2022**

**Lead societies:**

German Society for Epidemiology; German Society for Public Health; German Society of Paediatrics and Adolescent Medicine; and German Society for Paediatric Infectious Diseases

**Other member societies of the association of scientific medical societies:**

German Society for Social Paediatrics and Adolescent Medicine; German Society of Childhood and Adolescent Psychiatry and Psychotherapy; German Academy of Ethics in Medicine; Society of Hygiene, Environmental and Public Health Sciences; German Society for Social Medicine and Prevention; German Society for Hospital Hygiene; German Society for Virology; and German Society for Occupational, Social and Environmental Medicine

**Participation of further societies and organizations:**

Robert Koch Institute; Federal Association of Physicians of German Public Health Departments; Professional Association of Physicians in Child and Adolescent Medicine; State Health Department Baden-Wuerttemberg; Health Authority Nordfriesland; Health Authority Frankfurt am Main; Health Authority Neukölln; Standing Conference of Students; Children and Youth Advisory Committee of the German Children’s Fund; Child Protection Agency; Association for Education; General Association of Head Teachers Germany; Main Personnel Council for State Teachers at Comprehensive Schools, Rhineland- Palatinate; Association for Special Education; Federal Council of Parents; State Parent Council of Lower Saxony; State Parent Council of Saxony; Public Education Authority Cottbus; and German Educational Research Association,

**Observers:**

WHO Regional Office for Europe; Standing Conference of the Ministers of Education and Cultural Affairs; Authority for School and Occupational Training Hamburg; and Senator for Children and Education Bremen

SARS-CoV-2: severe acute respiratory syndrome coronavirus disease 2; WHO: World Health Organization.

Since regulations of the Association of Scientific Medical Societies require formal registration of the guideline by scientific societies, four such societies registered the school guideline with the association’s guideline register. Early on, the guideline secretariat, in collaboration with the association and the four scientific societies, made two critical choices, subsequently confirmed by all participating organizations. First, to facilitate evidence-informed decision-making, the guideline panel should develop an evidence-based guideline according to the association’s guidance for evidence- and consensus-based guidelines.^5^ This guidance includes forming a representative guideline group; managing conflict of interest; systematically searching and critically appraising evidence; formulating actionable recommendations and approving them in structured consensus conferences. Second, to ensure structured and transparent decision-making, the guideline panel should apply the WHO-INTEGRATE framework developed in cooperation with the World Health Organization (WHO).^8^ This evidence-to-decision framework adopts a whole-of-society perspective, emphasizing the consequences of recommendations beyond health.^9^ Therefore, the guideline secretariat chose this framework for this guideline because of the many unintended consequences of school measures beyond health, and its suitability for public health guidelines involving multiple sectors.

The WHO-INTEGRATE framework consists of six equally relevant criteria: balance of health benefits and harms; human rights and sociocultural acceptability; health equity, equality and non-discrimination; societal implications; financial and economic considerations; and feasibility and health system considerations, as well as the meta-criterion quality of evidence.^8^ Below we summarize the application of the framework during three key stages of guideline development, similar to applications of the framework in the development of WHO guidelines.^10^

### Scoping and stakeholder engagement

The whole-of-society perspective and multisectoral approach of the WHO-INTEGRATE framework supported the formal mapping of a broad range of stakeholders. This mapping led to the assembly of a guideline panel comprising societies and institutions representing health and education sectors, parent, teacher and student associations, as well as observers, including the WHO Regional Office for Europe (Box 1). During the first meeting, panellists agreed to focus on nine school measures: (i) cohorting and/or reducing the number of students in face-to-face teaching; (ii) wearing face masks; (iii) measures on the way to school; (iv) measures for music classes; (v) measures for physical education classes; (vi) dealing with suspected cases among students without known risk contact; (vii) dealing with contact individuals; (viii) ventilating classrooms; and (ix) using air purifiers.

### Informing research evidence needs

In contrast to usual procedures of guideline development in Germany, the first evidence syntheses started three months before the formal registration of the guideline in December 2020 (Fig. 1). Following registration, the guideline panel met for the first time and was able to use the initial results.^2^^,^^3^ Different versions of the living Cochrane rapid review of effectiveness of measures implemented in the school setting to contain the COVID-19 pandemic^2^^,^^7^ served as the up-to-date source of direct evidence (studies directly related to schools) in all versions of the school guideline. To identify indirect evidence (studies conducted in different populations and settings), the guideline secretariat conducted database searches for systematic reviews and reviewed reference lists of included publications and guidelines. The systems thinking of the WHO-INTEGRATE framework helped shape the Cochrane reviews, in particular during protocol development (for example, search terms, databases searches and eligible outcomes). The framework also emphasized the need for reviewing the unintended consequences of school measures. Due to the rapid nature of guideline development, evidence to support criteria beyond health benefits and harms was not systematically gathered for version 1.0 of the guideline; the Cochrane scoping review of unintended consequences^3^ with the potential to contribute direct evidence towards most WHO-INTEGRATE criteria, only became available from version 2.0.

### Developing recommendations

For eight school measures, one or two medical societies filled out the WHO-INTEGRATE evidence-to-decision table. For the ninth measure, the guideline secretariat completed the detailed table (online repository).^11^ One or two of the medical societies prepared a draft for each recommendation. The evidence-to-decision template made it straightforward to consider all WHO-INTEGRATE criteria explicitly, and influenced the type and wording of recommendations. All panellists received the recommendations for the nine measures, discussed them during a moderated consensus conference and revised, if necessary. The WHO-INTEGRATE criteria, where applicable, highlighted lack of alignment among panellists, often helping to reach consensus through slight reformulations. The guideline secretariat provided methodological support throughout the process, edited the recommendations and finalized the rationale for each measure, producing a visual presentation and a narrative overview (online repository).^11^

## Relevant changes

The guideline panel swiftly developed the school guideline under rapidly evolving conditions during the pandemic. The panel designed the guideline as a living document, allowing for updates to recommendations as soon as new relevant evidence became available. The first version was published in February 2021, four months after the first evidence syntheses were initiated and two months after the guideline’s official registration.

The launch of the first version of the guideline during a press conference by the German Minister of Research and Education in February 2021^12^ created much media attention.^13^ The guideline also gained international recognition.^14^ Through a carefully crafted dissemination strategy, the guideline was made readily available to health and education ministries at national and state levels.

We undertook an evaluation of the impact of the school guideline, contacting ministries of education in each state. Responses showed that almost all ministries were aware of the guideline, that the guideline was recognized as a relevant source of information in nine states, and that it was used to check existing directives in five states. We also conducted semi-structured interviews with individuals involved in school-related decision-making in two states, and with members of the guideline panel.^15^ All interviewees emphasized the value of the guideline given its evidence- and consensus-based development, but also noted limitations in applying the guideline across very different school contexts.^15^

## Lessons learnt

Using the WHO-INTEGRATE framework to develop a public health guideline under severe time constraints and with high political relevance offered a structured approach for considering a range of criteria from the start of guideline development. The framework was helpful in setting up the broad guideline panel, and the criteria were instrumental in showing alignment of views, surfacing sources of disagreement and guiding discussions towards consensus. The guideline panel appreciated the framework’s structured approach and its flexibility with regards to using both evidence and real-world expertise and experience. However, there was limited time for all panel members to fully engage with the framework (Box 2).

Box 2 **Summary of main lessons learnt**

- In Germany, an evidence- and broad consensus-based development process was essential for achieving high visibility and meaningful uptake of the living school guideline during the COVID-19 pandemic.
- The WHO-INTEGRATE framework facilitated a comprehensive assessment of school measures from the start of guideline development, considering their intended and unintended consequences from a whole-of-society perspective.
- Application of the WHO-INTEGRATE framework in rapid mode was feasible, more time for training guideline panellists and for discussing the criteria among the panel might increase the value added.

COVID-19: coronavirus disease 2019; WHO: World Health Organization.

The framework allowed for a comprehensive assessment of school measures, which was critical in view of their intended as well as unintended consequences. Criteria were assessed and weighed against each other, making use of the evidence available and the expertise represented. This structured approach allowed specific areas of contention to be identified and, in many cases, to be resolved. However, for several of the criteria beyond health benefits and harms, the lack of time and sufficient resources meant that evidence synthesis could not be conducted. For some of these criteria (for example, health equity, equality and non-discrimination), the panel could make a reasonable assessment given the expertise present. For other criteria the relevant expertise was missing (for example, legal aspects) or the assessment was deemed too complex (for example, macro-economic consequences).

Version 1.0 of the guideline was developed without dedicated funding as an emergency response during the pandemic. We estimate that evidence synthesis work to complete the initial scoping review and first version of the living rapid review over six months took at least 45 person-months. We estimate that guideline development required at least 20 person-months on behalf of the secretariat and coordinating body (approximately a quarter of this time was spent applying the WHO-INTEGRATE framework). Overall, the 32 contributing institutions invested at least 20 person-months. Sufficient resources for developing guidelines on school measures and other public health and social measures should be a core element of pandemic preparedness. 

The ability of the guideline development process to incorporate many different perspectives was well received in the media and among decision-makers. An evidence- and consensus-based development process helped achieve high visibility of the school guideline, nationally and internationally. We believe the WHO-INTEGRATE framework partly facilitated this largely positive reception. 

In conclusion, the WHO-INTEGRATE framework made the balancing of pros and cons of measures comprehensive, efficient and transparent. The use of the framework in rapid mode was feasible but fell short of its potential; more time for training guideline panellists and discussing the criteria would have been valuable.
